# The frequency of follicular T helper cells differs in acute and chronic neuroinflammation

**DOI:** 10.1038/s41598-020-77588-9

**Published:** 2020-11-24

**Authors:** Adalie Baniahmad, Katharina Birkner, Johanna Görg, Julia Loos, Frauke Zipp, Beatrice Wasser, Stefan Bittner

**Affiliations:** Department of Neurology, Focus Program Translational Neuroscience (FTN) and Immunotherapy (FZI), Rhine Main Neuroscience Network (rmn2), University Medical Center of the Johannes Gutenberg University Mainz, 55131 Mainz, Germany

**Keywords:** Neuroimmunology, Multiple sclerosis

## Abstract

Beyond the major role of T cells in the pathogenesis of the autoimmune neuroinflammatory disorder multiple sclerosis (MS), recent studies have highlighted the impact of B cells on pathogenic inflammatory processes. Follicular T helper cells (Tfh) are essential for the promotion of B cell-driven immune responses. However, their role in MS and its murine model, experimental autoimmune encephalomyelitis (EAE), is poorly investigated. A first step to achieving a better understanding of the contribution of Tfh cells to the disease is the consideration of Tfh cell localization in relation to genetic background and EAE induction method. Here, we investigated the Tfh cell distribution during disease progression in disease relevant organs in three different EAE models. An increase of Tfh frequency in the central nervous system (CNS) was observed during peak of C57BL/6 J EAE, paralleling chronic disease activity, whereas in relapsing–remitting SJL EAE mice Tfh cell frequencies were increased during remission. Furthermore, transferred Tfh-skewed cells polarized in vitro induced mild clinical symptoms in B6.Rag1^−/−^ mice. We identified significantly higher levels of Tfh cells in the dura mater than in the CNS both in C57BL/6 and in SJL/J mice. Overall, our study emphasizes diverse, non-static roles of Tfh cells during autoimmune neuroinflammation.

## Introduction

Multiple Sclerosis (MS) is a chronic autoimmune disease that is characterized by inflammation of the central nervous system (CNS) and neurodegeneration^1^. Pathogenic T helper (Th) cells, especially the subtypes Th1 and Th17, play a critical role in initiating and driving disease pathogenesis^2–4^. In light of the success of B cell targeting therapies such as ocrelizumab in clinical practice^5^, the role of B cells is currently being re-evaluated, and central questions about the interplay between T and B cells remain unclear. Moreover, the type II MS lesion pattern indicates a B cell-driven pathology in at least one subgroup of MS patients^6^.

In MS patients, B cells have been shown to form follicular structures in the meninges of patients, which seem to be linked to cortical grey matter damage^7–10^. The meninges are considered the connection between blood circulation and CNS parenchyma and can serve as both entrance and barrier to the nerval structures^11,12^. Due to their anatomic proximity to the CNS, the leptomeninges, including the arachnoidea and pia mater, have been the focus of studies discussing meningeal impact in neuroinflammation^10,13^. However, the latest research places great interest on the outermost meningeal layer, the dura mater, and its function as gateway to the periphery^11^.

Since the mouse model of MS, experimental autoimmune encephalomyelitis (EAE), can be provoked exclusively by T helper cells, research has been biased towards T cell-dependent pathogenesis. However, there are several possible B cell-specific functions that are thought to contribute to neuroinflammatory processes, and thus might have an effect on EAE progression. In their role as antigen presenting cells (APCs), B cells are known to participate in maintaining and aggravating T cell dependent autoimmune responses^14^. MCH II-dependent B cell activity is shown to play an important part in CNS autoimmunity^15^. Production by B cells of cytokines, such as IL-6^16^ or GM-CSF^17^, has a proinflammatory effect, whereas IL-10 reduces the inflammation process^18^. One important role of B cells in the immune system is the production of antibodies and an early indication of B cell participation in MS is the presence of oligoclonal bands in the cerebrospinal fluid (CSF) of MS patients. Indicating the presence of antibody-producing cells in the CNS, oligoclonal bands are one of the diagnostic criteria for MS^19^. Short-living plasma B cells were identified as the major B cell subpopulation involved in active inflammatory processes in MS patients^20^. Although the clinical success of plasmapheresis hints at a possible role of autoantibodies in MS pathogenesis^21^, a direct pathogenic role of autoantibodies in MS has so far not convincingly been shown.

Follicular T helper cells (Tfh), a CXCR5^+^ subgroup of T helper cells that can be found in the periphery in B cell follicles, have been described to promote B cell activity by supporting proliferation, antibody production, and class switching as well as follicle generation^22–24^. This indicates a possible impact of Tfh cells in autoimmune processes, such as in MS pathology.

Notably, several autoimmune-based diseases have been associated with the CXCR5^+^ subgroup of T helper cells, such as systemic lupus erythematosus^25,26^, rheumatic arthritis^27^ and Sjogren’s syndrome^28^ where they were thought to promote inflammation.

These findings suggest that Tfh cells may also be involved in MS progression. Nevertheless, there is still little evidence for a role of Tfh cells in MS pathology though investigation of Tfh cells in EAE gained more attention within the last years^29–32^. While it was shown that Tfh cells seem to aggravate ongoing inflammatory processes in EAE^29^, they do not induce disease symptoms on their own^31^. Nevertheless, possible phenotype changes or plasticity, which could contribute to any yet unknown Tfh interaction, has not been investigated in vivo.

Furthermore, it is still unclear whether Tfh cells play distinct roles in the development of chronic disease compared to a relapsing–remitting disease. A first step to gaining a better understanding of the contribution of Tfh cells to the disease is the consideration of Tfh cell localization during EAE.

In this work, we analyze the distribution of Tfh cells in different organs during the course of EAE. We further compare different models of disease progression: SJL/J to mimic relapsing–remitting MS (RRMS), and C57BL/6 EAE to mimic chronic disease. To get new insights into Tfh plasticity in vivo, we used B6.Rag1^−/−^ EAE as a passive EAE model to analyze their impact on the disease independent of the influence on other lymphocyte populations.

## Results

### Tfh cells are present in the dura mater of C57BL/6 EAE

Although it has been reported that Tfh cells are present in the CNS of EAE diseased C57BL/6 mice during acute inflammation^29,32^, Tfh cell distribution in central nervous structures during disease progression remained unclear. Since B cell follicles have been described in the meninges of MS patients and EAE mice^9^, we differentiated in our analysis between CNS (brain and spinal cord) and the outer layer of the skull meninges, the dura mater, for the examination of circulation dynamics.

**Figure 1:**
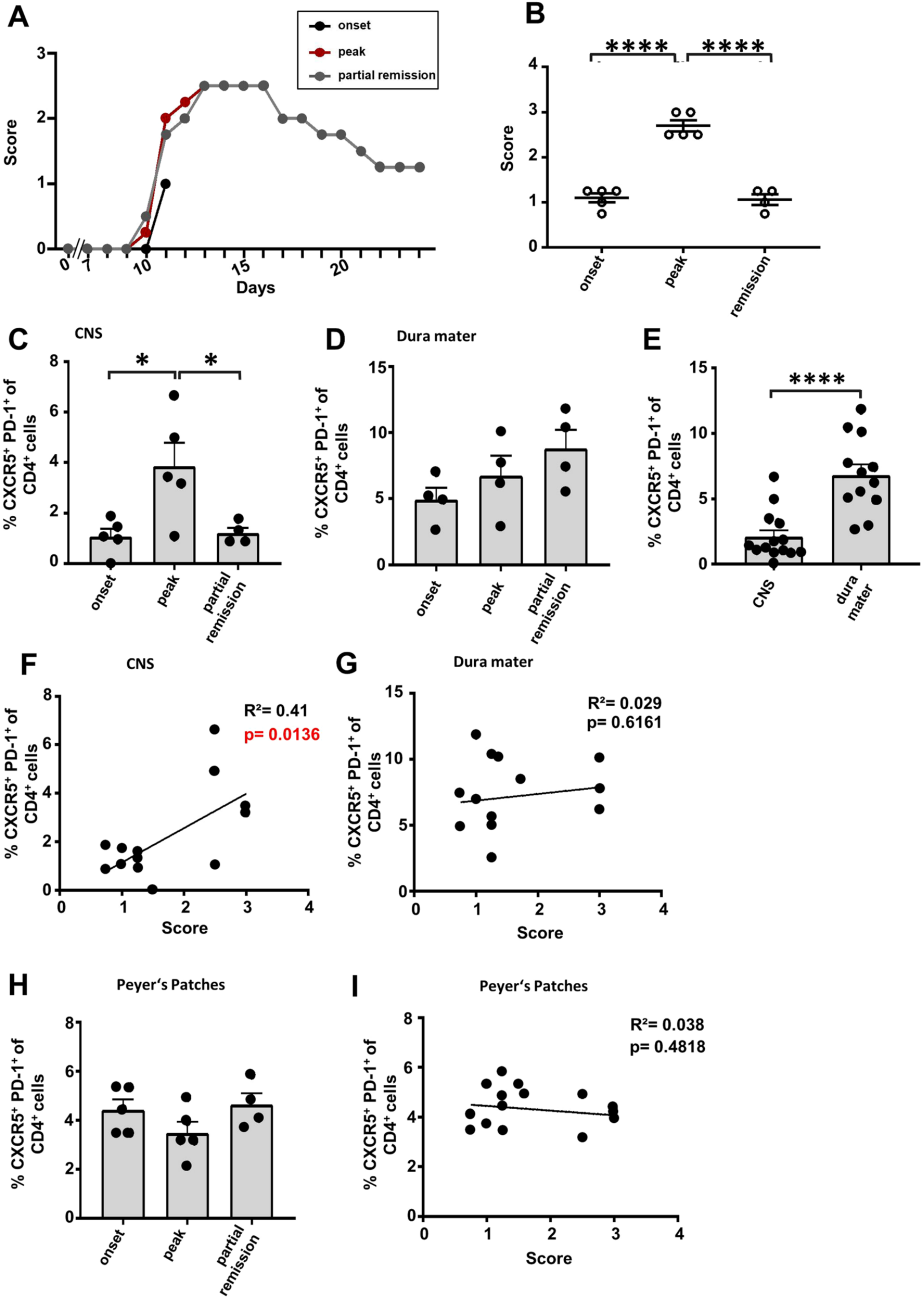
Tfh cell dynamics in C57BL/6 EAE. EAE was induced in C57BL/6 mice via MOG_35-55_ peptide immunization. (**A**) EAE course mimicking chronic disease progression, each stage represented by the disease progress of one exemplary mouse. Disease onset (dpi 10–12), peak (dpi 12–16) and partial remission (dpi 22–24) were defined dependent on the EAE course. The mean clinical score was compared between these different disease stages (**B**). (**C**–**E**) Percentage of Tfh cells (CXCR5 + PD-1 +) among T cells (living CD4^+^CD3^+^CD11b^−^CD45.2^+^ lymphocytes) were compared via FACS between the defined disease stages in the CNS (**C**) and dura mater (**D**). In addition, pooled Tfh frequencies from different time points (onset, peak, partial remission) of CNS and dura mater were compared (**E**). Data shown are mean ± SEM (**C**–**E**). (**F**, **G**) Correlation analysis between the percentage of Tfh cells and the clinical score of the CNS (**F**) and the dura mater (**G**). (**H** ,**I**) Analysis of the Tfh frequency (**H**) and the correlation analysis (**I**) in the Peyer’s patches. Results are representative of two independent experiments. Statistical analysis was performed using one-way ANOVA followed by Tukey’s multiple comparison test (**B**–**E**, **H**) or linear regression (**F**, **G**, **I**). *p < 0.05; **p < 0.01; ***p < 0.001; ****p < 0.0001. Onset n = 5, peak n = 5, partial remission n = 4.

We divided the disease course of MOG_35-55_ peptide-immunized C57BL/6 mice into three different stages, depending on the clinical score and the day post-immunization (dpi): onset (average dpi: 10–12; score-range: 0.5–1.25), peak (average dpi: 12–16; score range: 2.5–3) and partial remission (average dpi: 22–24; score range: 0–1.25) (Fig. 1A,B). As Tfh cells are a rather newly described T helper subpopulation, it lacks a common identification pattern. As previously reported^29,33,34^, the combination of CXCR5, PD-1, CD3 and CD4 emerge to be the main Tfh cell markers. To focus even more precisely on CD45^+^ leucocytes and prevent any contamination with CD11b^+^ monocytes and granulocytes, we characterized our target population as CD45.2^+^CD11b^−^CD3^+^CD4^+^CXCR5^+^PD-1^+^ living lymphocytes (Suppl. Figure S1). We saw a significant increase of Tfh cells in the CNS reaching its maximum at the peak disease stage, followed by a decrease in the remission phase (Fig. 1C). The identified Tfh cells expressed Bcl6 and IL-21 during all time points of the disease (Suppl. Figure S2). Importantly, we also found Tfh cells located in the dura mater (Fig. 1D and Suppl. Figure S2). Independent of disease stage, the Tfh frequency was higher in the dura mater than in the CNS (Fig. 1E). Altogether, our results suggest that a reservoir of Tfh cells persists in the dura mater, but Tfh cell alterations are pivotally associated with the degree of inflammation within the CNS, the target organ of the disease. Analyzing the absolute Tfh cell count confirmed significant Tfh cell accumulation both in the dura mater and in the CNS as compared to healthy controls (Suppl. Figure S3A). Of note, the frequency of Tfh cells correlated significantly with the disease score of EAE (p < 0.5) (Fig. 1F), while there was no significant correlation in the dura mater (Fig. 1G). These results suggest a pro-inflammatory role of Tfh cell frequencies in the C57BL/6-EAE. As recent studies have emphasized the role of the gut microbiota on MS/EAE pathogenesis^35^, as well as the impact of gut Tfh cells on other autoinflammatory diseases^36^, we further analyzed frequency changes in Peyer’s patches, representative for the secondary lymphatic tissue of the small intestine. Of note, despite strong differences in clinical disease severity (Fig. 1A,B), we did not detect any alterations of the Tfh cell frequencies in the Peyer’s patches (Fig. 1H), suggesting no effect of locally released factors of the microbiome on the Tfh compartment. We further did not detect any correlation of Tfh frequencies in the Peyer’s patches and clinical severity (Fig. 1I).

### Reduced Tfh cell frequencies in CNS with increased clinical score in SJL/J EAE

**Figure 2:**
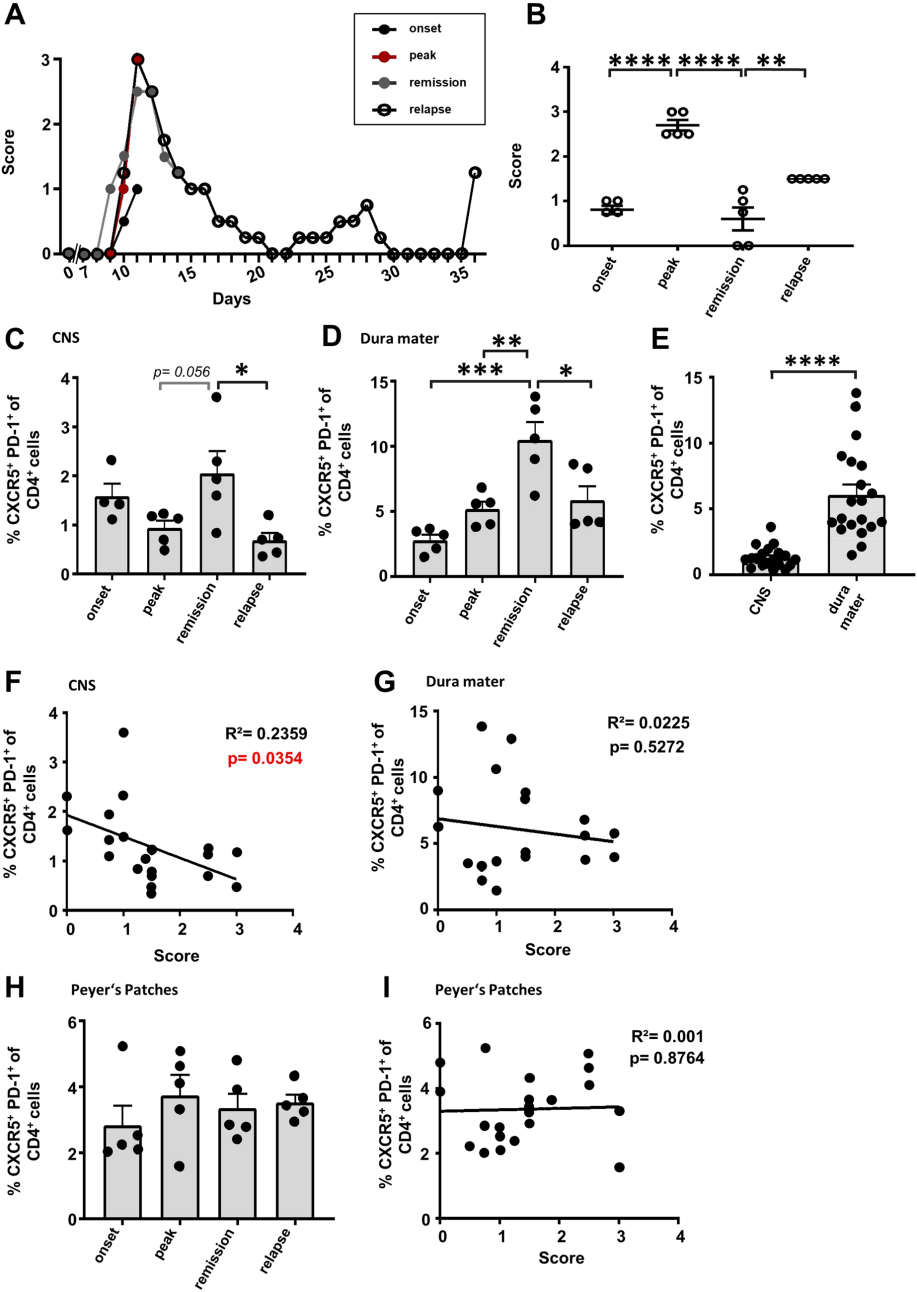
Tfh cell dynamics in SJL/J EAE. EAE was induced in SJL/J mice via PLP_139-151_ peptide immunization. (**A**) EAE course mimicking relapsing–remitting disease progression, each stage represented by the disease progress of one exemplary mouse. Disease onset (average of dpi: 9–11; score range: 0.5–1.25), peak (average of dpi: 11–13; score range: 2.5–3), remission (average of dpi: 16–17; score range: 0–1.25) and relapse (average of dpi: 34–36, score range: 1–2) of the disease were defined dependent on the EAE course. The mean clinical score was compared between these different disease stages (**B**). (**C**–**E**) Percentage of Tfh cells (CXCR5^+^PD-1^+^) among T cells (living CD4^+^CD3^+^CD11b^−^CD45^+^ lymphocytes) were compared between the defined disease stages in the CNS (**C**) and dura mater (**D**). In addition, pooled Tfh frequencies on different time points (onset, peak, partial remission and relapse) of CNS and dura mater were compared (**E**). Data shown are mean ± SEM (**C**–**E**). (**F**, **G**) Correlation analysis between the percentage of Tfh cells and the clinical score of the CNS (**F**) and the dura mater (**G**). (**H**, **I**) Analysis of the Tfh frequency (**H**) and the correlation analysis (**I**) in the Peyer’s patches. Results are representative of two independent experiments. Statistical analysis was performed using one-way ANOVA followed by Tukey’s multiple comparison test (**B**–**E**, **H**) or linear regression (**F**, **G**, **I**). *p < 0.05; **p < 0.01; ***p < 0.001; ****p < 0.0001. Onset n = 4, peak n = 5, remission n = 5, relapse n = 5.

Previous research on Tfh cells in EAE focused solely on the C57BL/6 J mouse model, which represents the chronic disease^29,32^. However, RRMS is a common form of the disease in humans. To get insights into Tfh cell involvement during RRMS, we used SJL/J mice to model this form of the disease. According to the disease progression of this model, we analyzed Tfh frequencies at onset (average dpi: 9–11; score range: 0.5–1.25), peak (average dpi: 11–13; score range: 2.5–3), remission (average dpi: 16–17; score range: 0–1.25) and relapse (average dpi: 34–36, score range: 1–2) of the disease (Fig. 2A,B).

Of note, the percentage of Tfh cells among CD4^+^ T cells in the CNS unexpectedly decreased during acute inflammation in the relapse of the disease compared to the remission phase (Fig. 2C). This effect could also be found in the dura mater, where a maximum in Tfh cell frequencies was observed during remission stage (Fig. 2D). Again, independent of disease stage, Tfh frequency was higher in the dura mater than in the CNS (Fig. 2E). Although the absolute Tfh cell count showed a tendency of reduced Tfh numbers in remission both in the CNS and in the dura mater (Suppl. Figure S3B), comparable to what was observed in the C57BL/6 model (Suppl. Figure S3A), we determined that Tfh cell frequencies among CD4^+^ T cells in the CNS showed a significant negative correlation to disease severity (Fig. 2F,G). Importantly, this is in contrast to the positive correlation seen in the chronic disease course of C57BL/6 mice, and indicative for an anti-inflammatory role of Tfh cell frequencies in the SJL/J-EAE. Together with the decreased Tfh frequency in SJL/J-CNS structures during acute inflammation, these results suggest opposing effects of Tfh cells during the different disease courses. Nevertheless, also in the SJL/J model, regulation of Tfh frequencies seemed to be especially important in the CNS. Comparable to the C57BL/6 model, no disease state-dependent alterations of Tfh cell frequencies were observed in the Peyer’s patches (Fig. 2H). Additionally, no correlation was detected between Tfh cell frequencies and the clinical score (Fig. 2I).

Collectively, these results indicate that Tfh cells may exert their effect on EAE disease progression locally within the affected CNS tissue with an opposite impact compared to the chronic C57BL/6 EAE model.

### Tfh-skewed cells are attained with a B cell co-culture in the presence of IL-21 and IL-6

**Figure 3:**
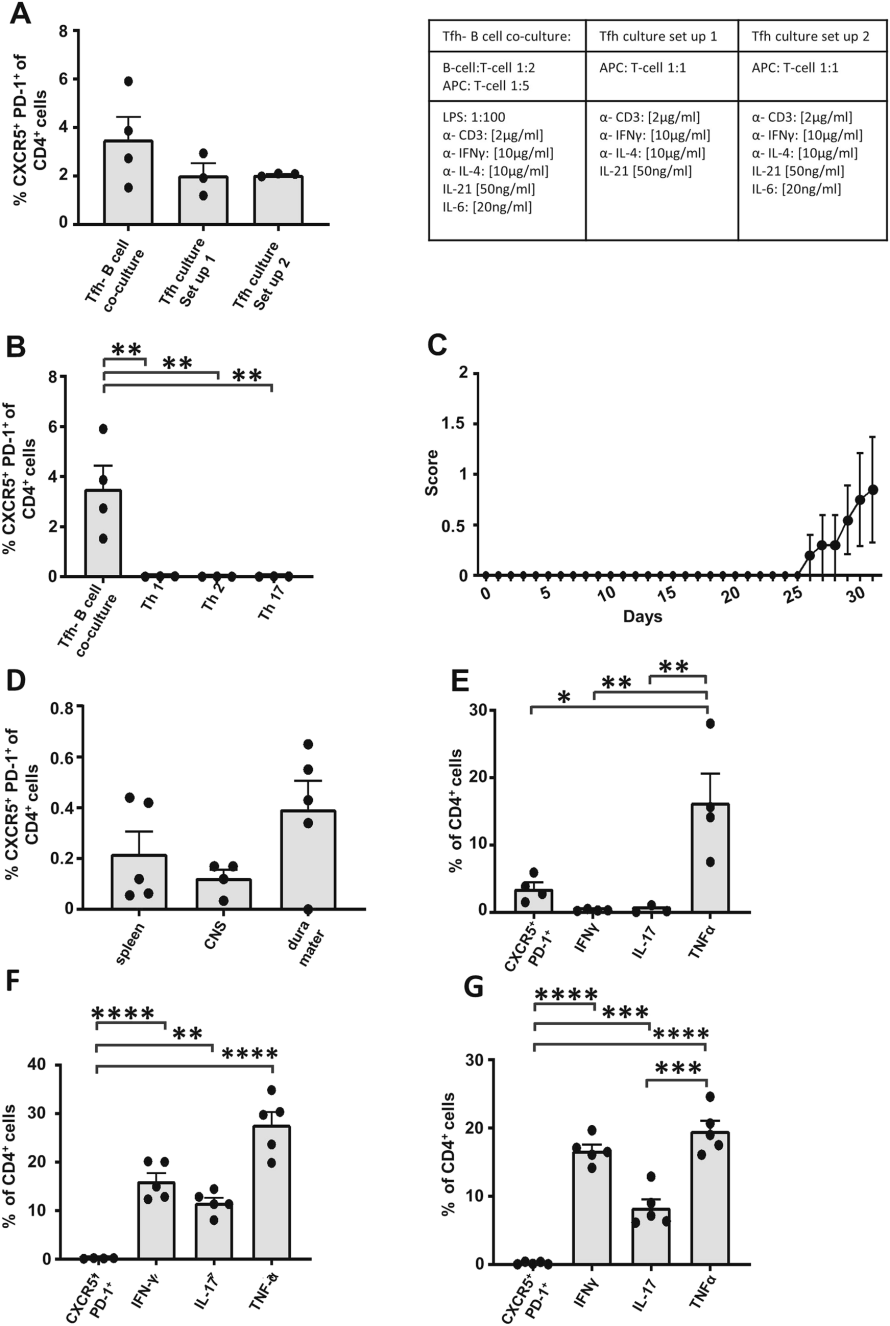
Transfer of in vitro cultured Tfh cells into Rag1^−/−^ mice. (**A**) Tfh cells were cultured in vitro under different conditions: Tfh-B cell co-culture with a B cell-T cell ratio of 1:2 and a CD90^−^ splenocytes—T-cell ratio of 1:5 in the presence of 2 µg/ml anti-CD3, 20 ng/ml IL-6, 50 ng/ml IL-20, 10 µg/ml anti-IL-4 and 10 µg/ml anti-IFNγ. Two different set ups of Tfh culture with a T-cell- CD90^−^ splenocytes ratio of 1:1 in presence of a similar cytokine mix. Set-up 1 in the absence of IL-6, set-up 2 in the presence of 20 ng/ml IL-6. (**B**) Flow cytometric analysis of Tfh cells (CXCR5^+^ PD-1^+^ of CD4^+^ cell) in Th1, Th2 and Th17 cultures in comparison to the Tfh-B cell co-culture. (**C**) EAE disease course of B6.Rag1^-/-^ mice intravenously treated with 5 × 10^6^ of the above described in vitro Tfh-skewed cell cultures (4% Tfh positive) per mouse. (**D**) Flow cytometric analysis of Tfh cell population in the spleen, the CNS and the dura mater was performed on dpi 31. (**C**–**G**) Analysis of the cytokine production (IFNγ, IL-17 and TNFα) and CXCR5^+^ PD-1^+^ expression of CD4^+^ lymphocytes in the in vitro Tfh culture on the day of transfer (**E**), compared to the lymphocytes CNS (**F**) and the spleen (**G**) of B6.Rag1^−/−^ EAE mice on dpi 31, n = 5. Statistical analysis was performed using one-way ANOVA followed by Tukey’s multiple comparison test (**A**, **B** ,**D**–**G**). *p < 0.05; **p < 0.01; ***p < 0.001; ****p < 0.0001. Tfh-B cell co culture n = 4, Tfh culture version 1 n = 3, Tfh culture version 2 n = 3, Th1 culture n = 3, Th2 culture n = 3, Th17 culture n = 3. Data shown are mean ± SEM.

In order to understand the function and plasticity of Tfh cells more precisely, we aimed to generate a polarized Tfh-skewed cell culture in vitro. Since the basis of this in vitro culture were magnetically sorted naïve T helper cells, the in vivo performed exclusion of CD11b^+^ cells was not necessary and we defined our target population to be CXCR5^+^PD-1^+^CD4^+^ T cells. Comparing different in vitro Tfh cell culture conditions^37,38^, or slight modifications of those cultures (Fig. 3A), we identified the B cell co-culture protocol previously published by *Kolenbrander *et al*. *^37^ but with modified cytokine concentrations (here, 2 µg/ml anti-CD3, 20 ng/ml IL-6, 50 ng/ml IL-21, 10 µg/ml anti-IL-4 and 10 µg/ml anti-IFNγ) and with a co-culture performed with CD90^-^ splenocytes instead of dendritic cells, to be most effective at generating CXCR5 + PD1 + cells (Fig. 3A), although overall frequencies remained rather low. In established in vitro cultures of Th1, Th2 and Th17 cells, we did not detect any differentiation into CXCR5^+^ PD1^+^ cells confirming this as a unique attribute of Tfh cells (Fig. 3B). For further characterization, this Tfh-skewed cell culture was transferred to B6.Rag1^−/−^ mice.

### Transfer of Tfh-skewed in vitro culture initiates EAE symptoms in B6.Rag1^−/−^ mice

Although Tfh cells during EAE gained attention recently, it is not known how stable in vitro-differentiated Tfh cultures are in vivo. After transferring the B6.2D2 Tfh-skewed culture (Fig. 3B) to B6.Rag1^−/−^ mice, we observed a late onset of EAE, leading to mild symptoms (Fig. 3C), thereby showing a lower capacity to induce EAE as compared to what is typically observed in adoptive transfer EAE after transferring pro-inflammatory Th17 cells (Suppl. Table 1). Because B6.Rag1^−/−^ mice lack intrinsic T cells and B cells^39^, we could exclude intrinsic T cell activity and that Tfh pathogenicity is solely mediated via B cell activation. Of note, Tfh frequencies were below 1% in all analyzed organs 31 days post immunization (Fig. 3D), showing that the Tfh phenotype was not stable in vivo. Similarly, compared to the cytokine levels in the in vitro culture on the day of injection (Fig. 3E), significantly increased levels of IFNγ, IL-17 and TNFα production were observed both in CNS and spleen (Fig. 3F,G), while Tfh frequency approached zero. Taken together, these results indicate that established protocols for in vitro-differentiation of Tfh cells yield Tfh cell cultures that show an unstable phenotype in vivo, thus conclusions regarding the in vivo-relevance of currently available in vitro-derived cultures have to be made with care.

## Discussion

Despite a proven influence of Tfh cells on autoimmune disease processes, Tfh impact on MS and the EAE model has gained increasing attention in recent years^29,32^. In the present study, we provide new insights into the role of Tfh cells in different models of EAE. We saw an increase in Tfh cells infiltrating the CNS in the inflammatory phases of C57BL/6 J EAE. Tfh cells play an important role in B cell activation^22–24^. Importantly, although MS is generally considered a T cell-triggered autoimmune disease, there is evidence for a contribution of B cells in neuroinflammation^5,40–42^. To date, the involvement of Tfh cells in EAE progression is, however, still highly debated and a lack of standardized approaches and markers for their investigation make functional analysis challenging. Initially described as a T helper subgroup in human tonsils expressing the B cell homing receptor CXCR5^22^, this population is now further characterized by expression of PD-1, IL-21 and Bcl6^34^. Since other cell lineages are able to express these features as well, an exact definition of the target population was a critical issue. Here, we prevented contamination with monocytes by excluding CD11b positive cells and focused on pure T helper cells by including only CD45^+^CD3^+^CD4^+^ cells into our analysis. To target our Tfh population as precisely as possible, we identified them as being PD-1 and CXCR5 double positive. The increase of Tfh cells in the CNS during inflammation reported here supports previous observations^29,32^ indicating a Tfh cell involvement in inflammatory mechanisms.

Since the role of the dura mater has been rarely included in investigations of inflammatory diseases despite its high content of vessels and lymphatics, we decided to put major focus on its analysis. As bridge between the periphery and central nervous structures, it can serve as gateway for inflammatory responses to access the CNS parenchyma. Although follicular structures were, until now, mainly described in the leptomeninges^10,13^ we found an enrichment of Tfh frequenices in the inflammatory disease stage in the dura mater, underlining its importance in neuroinflammation.

We also looked for the first time at Tfh cells in different EAE models that mimic different subtypes of MS^43,44^. Our data suggest an opposite Tfh cell dynamic during SJL/J EAE disease progression compared to C57BL/6 EAE. In the relapsing–remitting SJL-model, the highest Tfh frequency in the CNS was found in the non-inflammatory stage of remission with a drop in the relapse, suggesting that Tfh cells may support disease remission.

Reasons for different effects of Tfh cells in SJL/J and C57BL/6 EAE might be based on immunological variations between the mouse strains^45^. Interestingly, it has been shown that B cell/T cell ratios differ in SJL/J mice and C57BL/6 mice^46^. Whether B cell function also differs in SJL/J mice is poorly investigated. However, it is known that B cells can have conflicting roles in the immune response in neuroinflammation^47–49^. Although the B cell-depleting ocrelizumab leads to remission of MS symptoms, a phase two trial for B cell-depleting atacicept had to be terminated because of a high rate of relapses compared to the control group^50^. While the exact reason for the worsening is unknown, it was assumed that atacicept causes an incomplete reduction of B cells, which leads to an inequality of proinflammatory and regulatory B cells (Bregs). The IL-10-producing Bregs are a subgroup of B cells that are thought to have anti-inflammatory capacity, making them necessary for immune suppression and recovery in the mouse model EAE^49,51,52^.

We assume that the increased frequency of Tfh cells during remission in the CNS and dura mater of SJL/J EAE mice indicates a Tfh cell-mediated support of the anti-inflammatory regulatory B cell response, while the significant correlation of Tfh cells and clinical score in the CNS of C57BL/6 EAE mice suggests a promotion of pro-inflammatory B cell activity. As regulatory functions of B cells are known to be important for remission processes^53^, a predominant activation of Bregs in SJL/J mice could also underlie the stronger remission in SJL/J EAE as compared to C57BL/6 EAE.

Importantly, only the Tfh cell frequency but not the absolute Tfh cell count was increased during remission in SJL/J mice. The correlation of higher Tfh cell frequencies with reduced disease severity thus suggests that especially the relative number of Tfh cells as compared to other T helper cell subtypes is important in modulating the B cell response. Further studies are needed to elucidate how Tfh cells promote either pro- or anti-inflammatory B cell responses. As distinct distributions of B cell subtypes in the different disease courses could promote different inflammatory processes, it would be worth analyzing the differences in B cell dynamics in the distinct disease models. Identifying precise mechanisms of interaction between Tfh cells and the different B cell subtypes would be of great interest in order to uncover more specific targets for new therapeutic treatment strategies. In addition, it would be important to identify how Tfh cells are able to influence disease progression, independent of B cells. Here, we observed a switch from CXCR5^+^ PD-1^+^ Tfh-skewed cell cultures into IFNγ and IL17 producing CD4^+^ T helper cells. Despite the low number of transferred cells, this phenotype was sufficient to induce EAE in B6.Rag1^-/-^ mice. While Tfh cell plasticity^54^ may have led to a pro-inflammatory phenotype in vivo, Tfh cell decline with differentiation of potentially unpolarized naïve T helper cells into Th1 and Th17-like cells could have also resulted in central neuroinflammation. Altogether, our data indicate that improved culture conditions are needed to allow a more stable Tfh phenotype and thus clear interpretations of the effect of in vitro-skewed Tfh cells on the in vivo*-*disease phenotype.

However, our results in C57BL/6 and SJL EAE give new insights into the role and impact of Tfh cells in vivo in neuroinflammation and suggest new targets for basic research in the field of Tfh cells and B cells. In particular, we have identified different dynamics of Tfh cell frequencies in SJL/J EAE compared to C57BL/6 EAE suggesting that Tfh cells may be capable of both beneficial and detrimental effects during the disease, possibly leading to as-yet unexplored therapeutic approaches.

## Material methods

### Mice

Six- to eight-week-old female C57BL/6 J, B6.Rag1^−/−^^39^ and SJL/J mice were purchased from Janvier Labs (Saint-Berthevin Cedex, France) and bred in specific pathogen-free conditions. Mice were kept in-house in individually ventilated cages. All animal experiments were approved by local authorities (Landesuntersuchungsamt Rheinlandpfalz) and conducted in accordance to the German Animal Protection Law.

### Induction of experimental autoimmune encephalomyelitis (EAE)

Active EAE in C57BL/6 J mice was induced, as described before^55^, by subcutaneous four point immunization with 200 µg MOG_35-55_ peptide, which was either from Hook Kit EK 2210 according to manufacturer’s instructions or self-prepared. Additionally, 800 mg H37RA (Difco) emulsified in Complete Freund’s Adjuvant was given, followed by two applications of 200 ng pertussis toxin in PBS injected intraperitoneally at the time of immunization. Active EAE in SJL/J mice was induced similarly, using 250 µg PLP_139-151_ peptide (Hooke Laboratories) instead of the MOG peptide. For passive EAE, 5 × 10^6^ B6.2D2 Tfh-skewed cells were injected intravenously into B6.Rag1^−/−^ mice. Clinical signs of EAE were monitored daily and converted into clinical scores as described before^56^: 0: no detectable signs of EAE; 0.5: tail weakness; 1: complete tail paralysis; 2: partial hind limb paralysis; 2.5: unilateral complete hind limb paralysis; 3: complete bilateral hind limb paralysis; 3.5: complete hind limb paralysis and partial forelimb paralysis; 4: total paralysis of forelimbs and hind limbs; and 5: death.

Mice were anesthetized with 1.5% ketamine solution. The spleen was separated before perfusion and placed in RPMI media. In order to eliminate blood cells from the organs of interest, perfusion was performed by cutting the atrium and the vascular circulation was rinsed with a minimum of 20 ml PBS. Brain and spinal cord (CNS) were isolated in IMDM. The upper part of the skull was removed and placed in 5 ml PBS with 2% FCS. The small intestine was isolated, then the Peyer’s patches were cut off and collected in 5 ml washing media (RPMI media).

### Cell isolation of the CNS

Brain and spinal cord were cut into small pieces and digested for 30 min at 37 °C with collagenase IV (Sigma), DNAse I (Roche) and collagenase/dispase (Roche). Single cell suspension was achieved by filtering through a 100 µm cell strainer and resuspending in 5 ml 40% Percoll solution with IMDM. The suspension was placed on a 70% Percoll solution with PBS and centrifuged for 30 min at 750 g at room temperature with low acceleration and deceleration to generate a gradient. The fat layer was discarded whereas the cell layer between the Percoll and the IMDM phase was isolated.

### Cell isolation of the Peyer’s patches

The Peyer’s patches were mashed through a 100 µm cell strainer and centrifuged (500 g, 5 min, 4 °C). The cell pellet was resuspended in lysis buffer NH_4_Cl (8.29 g/l), KHCO_3_ (1 g/l), Na_2_EDTA (37.2 mg/l) in water) and washed.

### Cell isolation of the dura mater

The dura mater was gently pulled off of the upper part of the skull under the microscope, washed with PBS and digested in a digesting solution (Collagenase VIII (Roche), DNase1 (Roche), in PBS) for 30 min. This reaction was stopped with PBS + 2% FCS, and the suspension was homogenized by mashing through a 30 µm filter.

### Cell staining and flow cytometry

For surface staining, single-cell suspensions were prepared as described before. Staining was performed with Live-Dead viability-staining (propidium iodide or 7-Aminoactinomycin 7AAD (BioLegend) or fixable viability stain 450 (BD Biosciences)). For analysis of the murine cell phenotype, the following antibodies were used:

Anti-CD4-V450 (1:400) (clone: RM4-5, BD Bioscience), Anti-CD4-PeCy7 (1:1000) (clone: RM4-5, BD Bioscience), Anti-CD4-AF700 (1:200) (clone: RM4-5, BD Biosciences), Anti-CD3-APC (1:200) (clone: 1452-C11, BD Bioscience), Anti-IFNγ-V450 (1:200) (clone: XMG1.2, BD Bioscience), Anti-CD45.2- AlexaFluor 700 (1:400) (clone: 104, Thermo Fisher Scientific), Anti-CD45-BV605 (1:200) (clone: 30-F11, BD Bioscience); Anti-CD11b- Pe/Cy7 (1:200) (clone: M1/70, Thermo Fisher Scientific), Anti-CD11b-PerCP-Cy5.5 (1:200) (clone: M1/70, Biolegend), Anti-IL 17-APC (1:200) (clone: eBio17B7, Thermo Fisher Scientific), Anti-CXCR5-FITC (1:200) (clone: L138D7, BioLegend) and Anti-PD-1-PE (1:200) (clone: 29F.1A12, BioLegend), Anti-Bcl6-PECy7 (1:50) (clone: 7D1, Biolegend), Anti-IL21-APC (1:50) (clone: FFA21, Thermo Fisher Scientific).

Flow cytometry was performed with a FACS Canto II^57^ and data were analyzed using FlowJo 10.0 software (https://www.flowjo.com/solutions/flowjo).

### In vitro culture

B220^+^ B-cells and naïve CD4^+^CD62L^+^ cells were isolated and MACS-sorted from spleens and lymph nodes of B6.2D2 mice (6–10 weeks old), according to manufacturer’s protocol (both from MiltenyiBiotec), with a purity of > 93% of total cells. Murine T helper cell culture was performed similar to what was described previously^58^. In short, T cell differentiation was achieved in the presence of irradiated CD90^−^ splenocytes with the ratio of 1:5 (Th1, Th2) or 1:10 (Th17) and different cytokines. Th17 differentiation required 3 ng/ml huTGFβ (R&D Systems), 20 ng/ml IL-23 (R&D Systems), 20 ng/ml IL-6 (R&D Systems), 10 µg/ml anti-IL4 (R&D Systems), 10 µg/ml anti-IFNγ (BioXCell), and 2 µg/ml anti-CD3 (BD Bioscience).

Stimuli for Th1 differentiation were as follows: 50 ng/ml IL-12 (R&D Systems), 25 ng/ml IL-18 (MBL International), 10 µg/ml anti-IL4 (R&D Systems) and 2 µg/ml anti-CD3 (BD Bioscience). Th2 differentiation required 10 ng/ml IL-4 (R&D Systems), 10 µg/ml anti-IL12 (R&D Systems), 10 µg/ml anti-IFNγ (BioXCell) and 2 µg/ml anti-CD3 (BD Bioscience).

Differentiation into Tfh cells was modified from previous protocol^37^. Our cytokine compostition, containing 2 µg/ml anti-CD3 (BD Bioscience), 20 ng/ml IL-6 (R&D Systems), 50 ng/ml IL-21 (BioLegend), 10 µg/ml anti-IL-4 (R&D Systems) and 10 µg/ml anti-IFNγ (BioXCell), was added and plated with a ratio of 1:2 (B cells:T cells) on a 48-well plate at a density of 6 × 10^6^ cells per well. Irradiated CD90^-^ splenocytes instead of dendritic cells were added for initial stimulation in a ratio of 1:5 (CD90^−^ splenocytes:T cells). Cells were kept in cell culture medium for 5–10 days. Cytokine production was assessed using intracellular cytokine staining following standard protocols: anti-CD4-PeCy7 (1:1000) (clone: RM4-5, BD Bioscience), anti-IL-17A-APC (1:200) (clone: eBio17B7, Thermofisher), anti-IFNγ-Horizon (1:200) (clone: XMG1.2, BD Bioscience), anti-TNFα-AF700 (1:200) (clone: MP6-XT22BD, Bioscience) and anti-IL10- APC (clone: JES5-16E3, BD Bioscience). Surface staining was performed with CXCR5- FITC (1:200) (clone: L138D7, BioLegend), PD-1-PE (1:200) (clone: 29F.1A12, BioLegend).

### Statistics

All data were analyzed using GraphPad Prism 7 (GraphPad Software: https://www.graphpad.com/scientific-software/prism/) if not otherwise declared. Mean group differences were examined by one-way ANOVA followed by Tukey’s multiple comparison test or independent-sample t-tests. Data are presented as the mean ± standard error of the mean (SEM) unless otherwise indicated. To identify and exclude outliers, the ROUT method enclosed in GraphPad was used. P values < 0.05 were considered statistically significant.

## Supplementary information

Supplementary Information 1.
