# Supplementary material for: The frequency of follicular T helper cells differs in acute and chronic neuroinflammation
Source: Sci Rep. 2020 Nov 24;10:20485. doi: 10.1038/s41598-020-77588-9 (PMC7686332; doi:10.1038/s41598-020-77588-9)
Supplement: Supplementary file 1 — Supplementary Information 1. [file 41598_2020_77588_MOESM1_ESM.pdf]

The frequency of follicular T helper cells differs in acute and chronic neuroinflammation

Adalie Baniahmad, Katharina Birkner, Johanna Görg, Julia Loos, Frauke Zipp, Beatrice Wasser, and Stefan Bittner

Supplemental Material

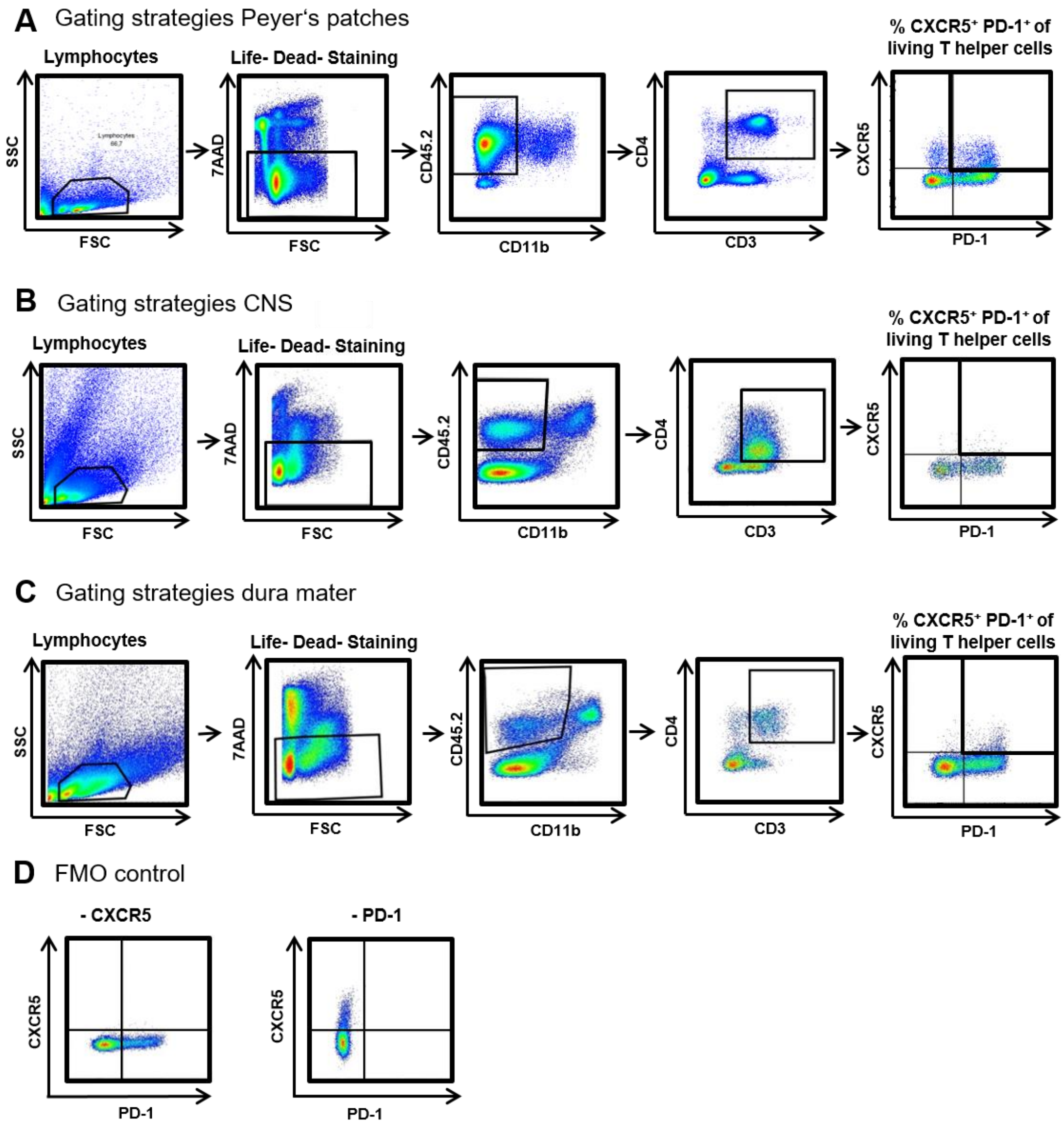

Supplemental Figure 1: Gating strategies of Tfh cells in flow cytometry analysis. Single cell suspensions of Peyer's patches **(A)**, CNS **(B)** and dura mater **(C)** of EAE-diseased SJL/J and C57BL/6 mice were prepared and analyzed by FACS. Live-dead-staining of the lymphocytes was performed with 7-aminoactinomycin (7AAD). The inclusion of leucocytes and exclusion of monocytes followed, gating on the CD45+ and CD11b- population. T helper cells were then defined as CD4+ CD3+ leucocytes. Finally, our target population of Tfh cells was characterized as the percentage of CXCR5+ PD-1+ cells of living T-helper cells. Additionally, Fluorescence Minus One (FMO) control for the relevant marker CXCR5 and PD-1 was performed **(D)**.

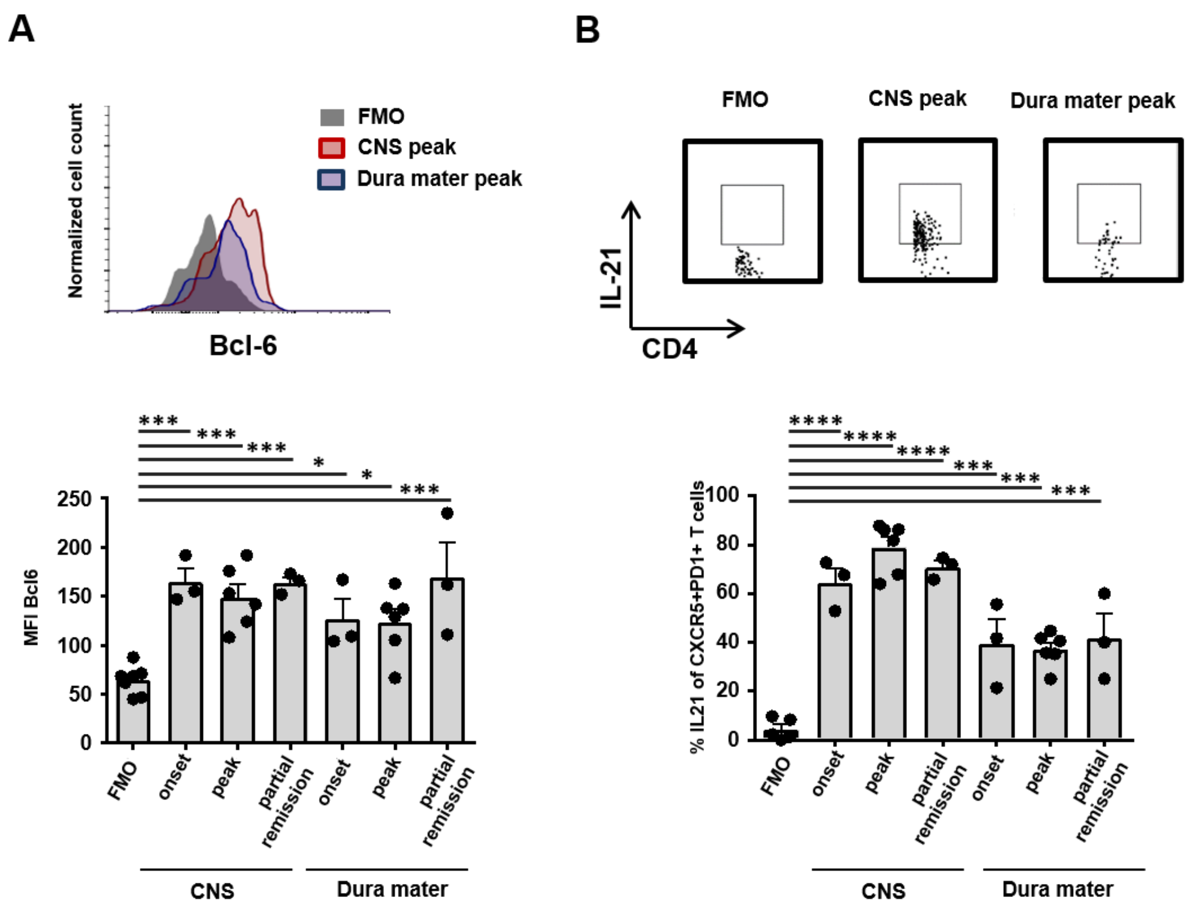

Supplemental Figure 2: Bcl6 and IL-21 are expressed by all analyzed Tfh cells. EAE was induced in C57BL/6 mice via $MOG_{35\text{-}55}$ peptide immunization. **(A)** Mean fluorescence intensity (MFI) of Bcl6 in living CD45+CD11b-CD4+CXCR5+PD-1+ cells is shown representatively during the peak of the disease in the CNS (red) and the dura mater (blue) in histograms (upper panel) and quantified in comparison to fluorescence minus one (FMO)-controls during onset (n=3), peak (n=6) and remission (n=3) both in the CNS and in the dura mater of EAE-diseased mice. **(B)** Percentages of IL-21 in living CD45+CD11b-CD4+CXCR5+PD-1+ cells is shown representatively during the peak of the disease in the CNS and the dura mater (blue) in dot plots (upper panel) and quantified in comparison to FMO-controls during all onset (n=3), peak (n=6) and remission (n=3) both in the CNS and in the dura mater of EAE-diseased mice. Statistical analysis was performed using one-way ANOVA followed by Dunnett's multiple comparison test. * $p<0.05$; *** $p< 0.001$; **** $p< 0.0001$.

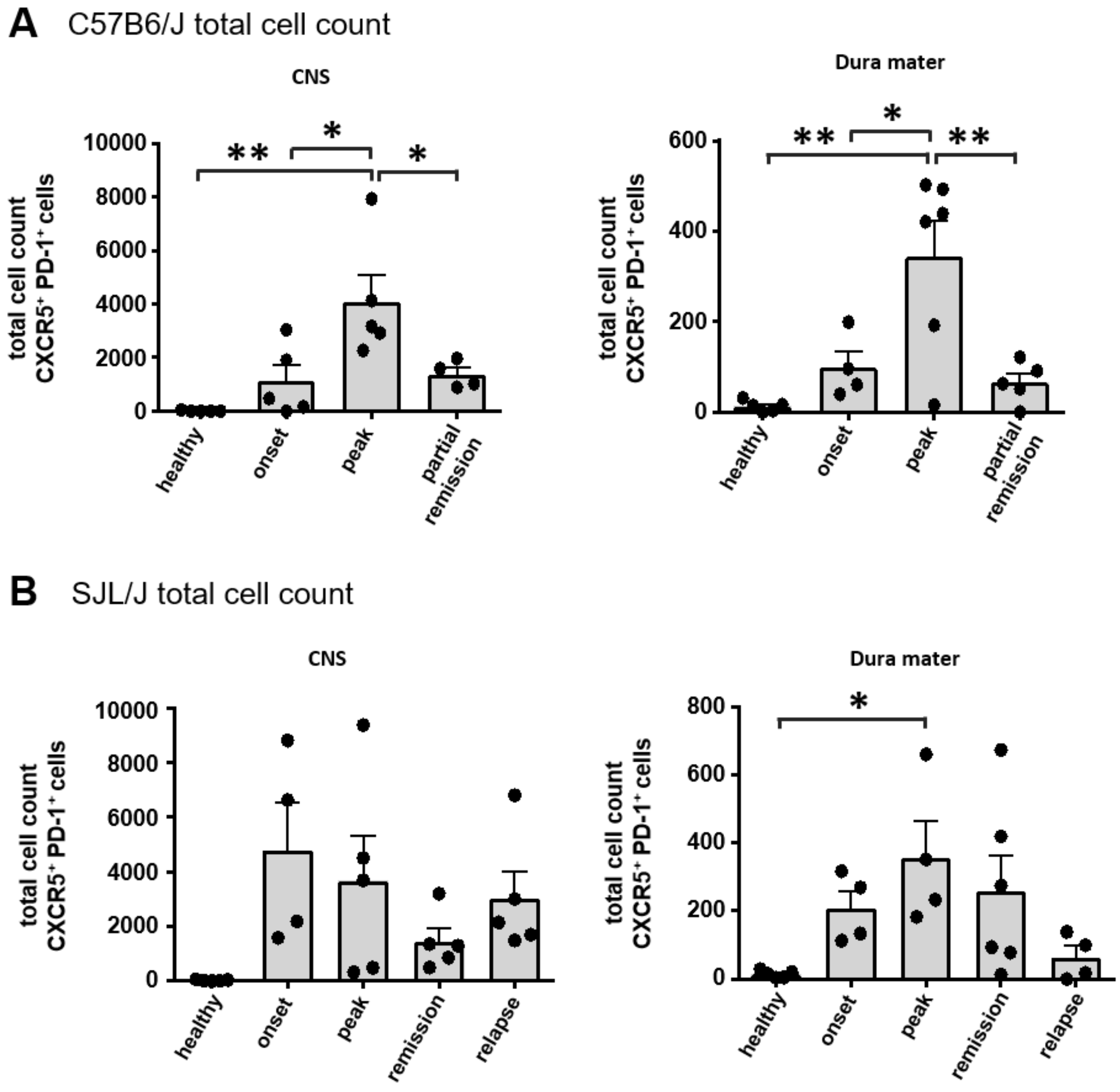

Supplemental Figure 3: Absolute Tfh cell count in health and disease. EAE was induced in C57BL/6 mice via $MOG_{35\text{-}55}$ peptide immunization **(A)** or in SJL/J mice via $PLP_{139\text{-}151}$ peptide immunization **(B)**. In addition, non-immunized (healthy) mice were analyzed. Absolute numbers of living Tfh cells (CD45+CD11b-CD4+CXCR5+PD-1+) were compared between the defined disease stages (n=4-5) in the CNS (left panel) and dura mater (right panel). Statistical analysis was performed using one-way ANOVA followed by Tukey's multiple comparison test. * $p<0.05$; ** $p< 0.01$.

Supplemental Table 1: Characteristics of Th17 cell adoptive transfer EAE.

Characteristics of adoptive EAE: Th17-skewed cells transferred into Rag2-/- mice	
**sex of mice (f/m)**	20/3
**total mice included**	23
**day of onset (mean + range)**	19.5 [18-22] days
**day of peak (mean + range)**	26 [24-30] days
**time between onset and peak (mean + range)**	6.5 [3-12] days
**maximum score (mean + range)**	3.25 [3-4]
